# Evaluating Theory-Driven Messaging to Overcome the Barriers to Meditation: Large-Scale Digital Field Experiment

**DOI:** 10.2196/71732

**Published:** 2025-07-07

**Authors:** Michael Bowen, Michael Beam, Joakim Semb, Dong Whi Yoo

**Affiliations:** 1 Kent State University, 1125 Risman Dr., Kent, OH, 44242, United States; 2 Spotify (Sweden), Stockholm, Sweden

**Keywords:** meditation, mindfulness, barriers, self-efficacy, experiment, messaging, health communication, mental health, app, mobile app

## Abstract

**Background:**

The general public is largely aware of meditation, and there is compelling evidence the practice has health benefits. But many people who are aware of meditation have not tried it, and those who do often struggle to establish a regular practice. The barriers to meditation are generally understood and include a lack of knowledge, a lack of time, and unclear benefits. These barriers present an impediment to self-efficacy in establishing a meditation practice. Despite these challenges, current strategies for promoting meditation may fail to address these barriers, leaving a gap in our knowledge about health communication efforts aimed at fostering meditation practices.

**Objective:**

The objective of this research is to leverage a large-scale, real-world digital platform to understand whether breaking down the theory-based barriers to meditation can serve as an effective strategy for encouraging meditation.

**Methods:**

This research is a digital messaging-based experiment that includes approximately 1.33 million people, aged 18 years and older, in the United States. The experiment was conducted on the Spotify mobile app and includes 1 control condition and 4 test conditions. Each of the test conditions was a message that attempted to address a specific barrier to meditation. The control message only included the call-to-action without any theory-based messaging accompaniment. When users clicked the message, they were redirected to meditation content. The click-through rate and the activation rate of each message were the dependent variables in the experiment.

**Results:**

The most effective message, which was designed to break down the pragmatic barriers to meditation, had a click-through rate odds ratio (OR) of 1.57 (95% CI 1.52‐1.62) and an activation rate OR of 1.55 (95% CI 1.45‐1.65), relative to the control. The least effective, which was designed to break down knowledge barriers, had a click-through rate OR of 0.91 (95% CI 0.88‐0.94) and an activation rate OR of 0.66 (95% CI 0.61‐0.71), compared to the control. After 7 days, the differences in people’s engagement with the meditation content itself between experimental conditions had substantively diminished.

**Conclusions:**

Theory-driven messaging can potentially encourage people to explore meditation content, but not universally so, given 2 of the experimental conditions performed better than and 2 performed worse than the control. The most successful message broke down the barrier that meditation requires being alone and in a quiet place. Addressing this barrier may have boosted self-efficacy by aligning the practice with everyday settings that fit into people’s busy lifestyles. Future researchers might consider how to encourage people to engage in meditation during their daily activities. In addition, breaking down the barriers to meditation through messaging can drive interest and experimentation with meditation content, but may not be enough to compel meaningful behavior change.

## Introduction

### Background

The practice of meditation has gained popularity for its mental health benefits, including reducing stress and anxiety, managing emotions, and enhancing happiness and overall life satisfaction, among others [1-7]. There is also compelling evidence that meditation offers mental health benefits in both clinical and nonclinical settings, including individuals with psychiatric disorders as well as those without [8-11].

Despite the clear benefits, the adoption and maintenance of meditation practices have low engagement. Recent research revealed that while 92% of Americans are aware of meditation, less than half (49.8%) have ever tried it, and only 15.1% have engaged in the practice within a 7-day time period [12]. Meditation content is also widely available on digital platforms, such as Calm, Headspace, YouTube, and Spotify. Among meditators, 58.8% have used meditation apps at least once [13]. However, retention with meditation apps is low. In an analysis of 29 mindfulness meditation apps, the median 7-day retention rate was just under 10% and the median 30-day retention rate was about 5% [14]. This data highlights a disconnect between awareness and sustained practice, indicating the importance of understanding the barriers to engagement with meditation.

Along these lines, past research has systematically identified the barriers to meditation. Williams et al [15] developed a 17-item survey battery that explored the self-reported barriers to meditating. These barriers were further refined, and the dimensionality was reduced by Hunt et al [16], resulting in four primary types of barriers to meditation (Textbox 1):

Textbox 1. **Perceived barriers to meditation**

- Perceived pragmatic barriers: Practical issues such as finding a quiet place or carving out time exclusively for meditation present logistical challenges. These can be particularly daunting for those with demanding schedules or limited private space.
- Low perceived benefits: Many individuals view meditation as unproductive or a waste of time. This perception stems from a misunderstanding of the practice and can deter individuals who prioritize short-term, immediately tangible benefits.
- Perceived sociocultural conflicts: Sociocultural perceptions can also deter individuals from meditating. For example, if family members or peers view meditation as unconventional or strange, the individual might feel dissuaded from practicing due to fear of social ostracism or misunderstanding.
- Perceived inadequate knowledge: A common barrier is the lack of knowledge about how to meditate properly. Potential meditators may feel uncertain about the “right” way to engage in meditation, fearing that incorrect practices might make the effort unproductive.

Understanding and breaking down these perceived barriers is important in increasing people’s self-efficacy, a central construct in social cognitive theory (SCT) [17]. Abraham and Mitchie [18] additionally identified barrier prompting as a component to testing SCT. This is the theoretical basis we used in conducting this research. In doing so, breaking down the barriers to meditation may increase people’s self-efficacy in practicing meditation, and even subsequently adopting a meditation habit. This could ultimately lead to better mental health outcomes for more people.

### Research Purpose

The purpose of this research is to leverage a large-scale field experiment on a digital platform to explore whether addressing the barriers to meditation through messaging can generate interest in engaging with meditation content. In doing so, this study uses both the click-through rate (the percentage of people who clicked on the message after having been presented with the message) and the activation rate (the percentage of people who began playing the content) to assess the effectiveness of each message. With over 1.33 million people included in the experiment, this research aims to examine these research questions and hypotheses (Textbox 2).

Textbox 2. **Research question and hypotheses.**

- Research question: Are targeted messages that specifically address the theoretical barriers to meditation more effective in encouraging individuals to engage with meditation content compared to generic promotional messages?
  - Hypothesis 1a: Attempting to break down the perceived pragmatic barrier to meditation will achieve a higher click-through rate than the control.
  - Hypothesis 1b: Attempting to break down the perceived pragmatic barrier to meditation will achieve a higher activation rate than the control.
  - Hypothesis 2a: Attempting to break down the low perceived benefits barrier to meditation will achieve a higher click-through rate than the control.
  - Hypothesis 2b: Attempting to break down the low perceived benefits barrier to meditation will achieve a higher activation rate than the control.
  - Hypothesis 3a: Attempting to break down the perceived sociocultural conflict barrier to meditation will achieve a higher click-through rate than the control.
  - Hypothesis 3b: Attempting to break down the perceived sociocultural conflict barrier to meditation will achieve a higher activation rate than the control.
  - Hypothesis 4a: Attempting to break down the perceived inadequate knowledge barrier to meditation will achieve a higher click-through rate than the control.
  - Hypothesis 4b. Attempting to break down the perceived inadequate knowledge barrier to meditation will achieve a higher activation rate than the control.

By examining these hypotheses, this research may also help future researchers who study meditation understand what compels people to engage with, explore, and experiment with digital meditation content. In addition, the research can be used to better understand the impact of addressing barriers to health behavior change through messaging and subsequently further refine theoretical models. Finally, this study provides empirically validated insights that meditation program managers and content providers can use to develop compelling messaging that will more effectively attract people to the practice.

## Methods

### Sampling and Recruitment

This research used a messaging-based experimental design that included 1,328,865 Spotify Premium users, aged 18 years and older, in the United States. Each person in this population viewed one of the messages across conditions. Refer to Table 1 for the sample sizes, as well as the age and gender composition of the sample for each condition. Note that date of birth and gender are collected during the Spotify registration process.

As the primary experimental intervention, participants across all conditions were assigned to receive one of 5 potential messages to encourage them to listen to meditation content. Four of the messages were test conditions, and one was the control condition. The messages were served in the Spotify mobile app and were triggered when people in the sample visited the app’s homepage. The message type and call-to-action from this campaign are similar to the various types of in-app messages and notifications that Spotify users are accustomed to receiving. People who received the message could choose to click the message call-to-action or dismiss the message. By clicking on the message, participants were redirected to an introductory meditation program on Spotify. The introductory program consisted of 14 total meditation sessions and lessons, ranging from about 3 minutes to about 11 minutes in length. Data collection was conducted on March 31 and April 1, 2024.

**Table 1.** Total sample, age, and gender composition by condition.

	Condition 1 (control)	Condition 2	Condition 3	Condition 4	Condition 5
Total sample^a^, n[Table-fn T1_FN1]	205,274	280,709	280,106	281,390	280,905
Age, %					
18‐24	32.50	33.51	33.75	33.59	33.57
25‐34	33.94	33.59	33.58	33.62	33.63
35‐44	18.25	18.04	17.85	17.87	18.06
45‐54	9.00	8.83	8.78	8.79	8.78
55+	6.31	6.02	6.04	6.13	5.97
Gender identification, %					
Female	45.18	45.34	45.20	45.22	45.29
Male	52.33	52.14	52.27	52.21	52.18
Other, not listed or prefer not to say	2.42	2.46	2.48	2.51	2.47

a The messaging system sent fewer messages to the control condition, but this did not appear to introduce a systematic bias to the experiment.

### Experimental Design

Each of the test conditions was a brief message that attempted to address one of the specific barriers to meditation that was identified by Williams et al [15] and later refined by Hunt et al [16]. The control message only included the call-to-action without any theory-based messaging accompaniment.

Condition 1 was the control message and was not theory-based. It was designed to inform people that the content was available on Spotify. The headline copy for the message was “Try Practicing Meditation On Spotify” with the body copy “Click below to get started with meditation content on Spotify.” The word count for this message is 15. To further assess the readability of the messages included in the experiment, the Flesch Reading Ease Score was employed [19]. The Flesch Reading Ease Score uses a 100-point scale, in which the higher the score, the more comprehensible the text. To calculate this score, the Textstat Python library was used [20]. The Flesch Reading Ease Score for Condition 1 is 30.0.

The message in Condition 2 was designed to break down the perceived pragmatic barriers to meditation—specifically that people do not meditate because there is no quiet place to do so or there is never time when they can be alone to meditate [16]. The headline copy for the message was “To Meditate, You Don’t Need Silence” and the body copy “Meditation can happen wherever you are, not just in quiet places when you are alone. Click below to get started with meditation content on Spotify.” The word count for this message is 31 and the Flesch Reading Ease Score is 61.0.

The Condition 3 message was designed to provide high-level information that meditation has many potential benefits, which addresses the low perceived benefit barriers identified by Hunt et al [16], including that meditation does not accomplish anything and that it is a waste of time to sit and do nothing. The message headline is “Meditating For Just A Few Minutes A Day Can Transform Your Mind” and the body copy “Practicing meditation can have numerous potential benefits. Click below to get started with meditation content on Spotify.” The word count for this message is 29 and the Flesch reading ease score is 44.7.

Condition 4 was designed to address the perceived sociocultural conflict barrier that others might think it is unusual to meditate [16]. The message headline was articulated as “Join The Millions Of People Who Are Meditating Today” with the body copy “Meditation has become popular around the world. Click below to get started with meditation content on Spotify.” The word count for this message is 26 and the Flesch Reading Ease Score is 45.7.

Finally, Condition 5 was designed to address the perceived inadequate knowledge barrier to meditation, including not knowing much about meditation and not knowing whether one was meditating correctly [16]. The headline copy was articulated as “Learn How To Meditate With Expert Practitioners” with the body copy “You can learn effective meditation techniques from expert teachers. Click below to get started with meditation content on Spotify.” The word count for this message is 26 and the Flesch Reading Ease Score is also 45.7.

**Figure 1.**
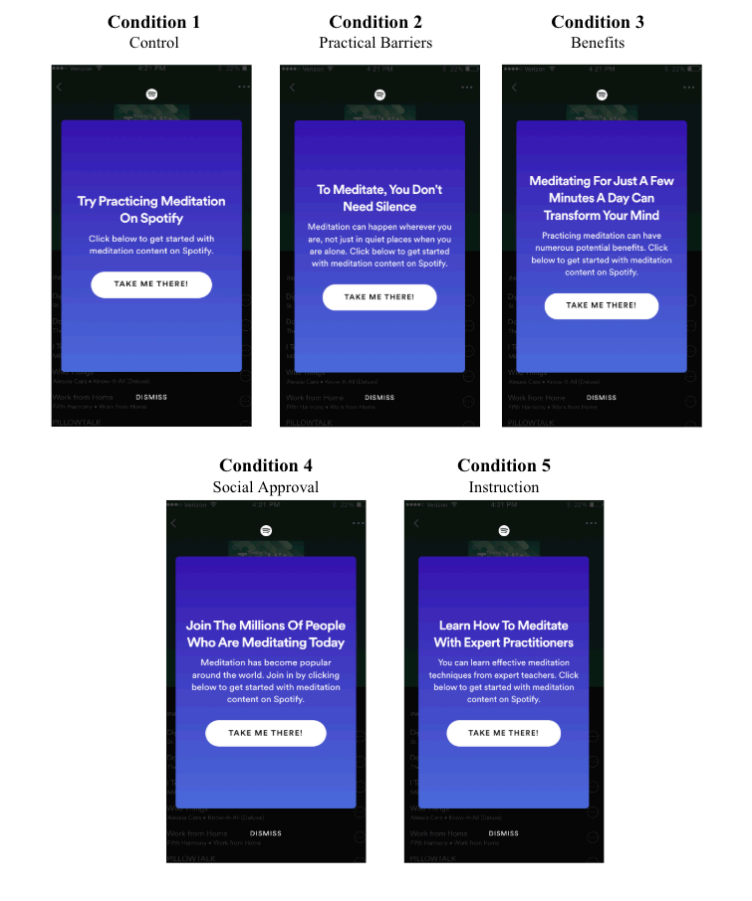
Experimental design messaging copy by condition.

Figure 1 provides a summary of the specific messages, including the message headline and body copy, that were used in each condition.

### Data Analysis

The analysis was conducted using Python. Only people who viewed the respective message they were assigned were included in the analysis, totaling about 1.33 million across all conditions. The percentage of people who clicked on the messages, otherwise known as the click-through rate, was the primary dependent variable in the experiment used to test hypotheses 1a, 2a, 3a, and 4a. This was chosen because the measure can represent an expression of interest in the meditation content based on the messaging provided. In addition, the activation rate, or the percentage of people in each condition who began playing the content itself, was the primary dependent variable used to test hypotheses 1b, 2b, 3b, and 4b. This was chosen because the measure represents an interest in exploring the content itself. A *χ*^2^ test of independence was initially used to compare each of the click-through rates and activation rates across all conditions.

To explore the effect size of each condition relative to the control, we used logistic regression, using the statsmodels Python package [21]. Two models were developed. The first model used click-through rate as the dependent variable, and the second model used the activation rate as the dependent variable. Each condition was inserted separately as an independent variable in each respective model, and the control condition was held out as the reference category. In addition, age and gender were included as independent variables in each model. Age was binned into 5 groups and one-hot encoded as 18‐24, 25‐34, 35‐44, 45‐54, and aged 55 and older. 18‐24 was held out of the model as the reference category. Gender values included female, male, and other, and were also one-hot encoded for modeling. Male was held out as the reference category.

### Ethical Considerations

The study was reviewed and approved by the Kent State University Institutional Review Board (IRB) under IRB reference number 1251. The consent process was governed by Spotify’s terms of service and privacy policy, as the experiments were conducted on the platform with its members. The research team and the IRB carefully reviewed these policies and the ethical conduct of the study, concluding that consent was obtained through the terms of service and privacy policy. The IRB also determined that this study qualifies as a benign behavioral intervention. Personally identifiable information about Spotify users was not accessible to the researchers and is safeguarded through encryption. It can only be accessed through a review and approval process by the Spotify Data Protection Office.

## Results

### Overview

The *χ*^2^ test for the click-through rate across all conditions yielded *χ*^2^_4_=2156.85 (*P*<.001), indicating significant differences in click-through rate across conditions. The *χ*^2^ test for the activation rate across all conditions yielded *χ*^2^_4_=1037.73 (*P*<.001), also indicating significant differences in activation rate across conditions.

In the logistic regression to assess the differences in effect size of the click-through rates, Condition 2, which addressed the perceived pragmatic barriers to meditation, had the largest effect (odds ratio [OR] 1.57, 95% CI 1.52‐1.62), followed by Condition 3, which highlighted that there are benefits to meditation (OR 1.25, 95% CI 1.21‐1.29).

Conditions 4 and 5, which were designed to address the perceived sociocultural conflicts and the perceived inadequate knowledge about meditation, each performed worse than the control on the click-through rate, respectively (OR 0.92, 95% CI 0.89‐0.95; OR 0.91, 95% CI 0.88‐0.94). Refer to Table 2 for more details on the logistic regression model of the click-through rate.

**Table 2.** Odds ratio (OR), coefficient, SE, and *P* value from logistic regression of click-through rate.

	OR (95% CI)	Coefficient	SE	*P* value
Constant	0.02 (0.021-0.023)	–3.787	0.02	<.001
Experimental condition				
Control	*—^a^*	*—*	*—*	*—*
Condition 2	1.57 (1.52-1.62)	0.450	0.02	<.001
Condition 3	1.25 (1.21-1.29)	0.225	0.02	<.001
Condition 4	0.92 (0.89-0.95)	–0.085	0.02	<.001
Condition 5	0.91 (0.88-0.94)	–0.096	0.02	<.001
Age group (years)				
18‐24	*—*	*—*	*—*	*—*
25‐34	1.21 (1.19-1.24)	0.194	0.01	<.001
35‐44	1.53 (1.49-1.57)	0.424	0.01	<.001
45‐54	1.50 (1.45-1.56)	0.408	0.02	<.001
55+	1.42 (1.36-1.48)	0.349	0.02	<.001
Gender identification				
Female	1.16 (1.14-1.18)	0.149	0.01	<.001
Male	*—*	*—*	*—*	*—*
Other	1.34 (1.25-1.42)	0.290	0.03	<.001

a Not applicable.

In the logistic regression to assess the differences in effect sizes of the activation rate, Condition 2 similarly performed best (OR 1.55, 95% CI 1.45‐1.65), followed by Condition 3 (OR 1.38, 95% CI 1.29‐1.47). Conditions 4 and 5 also underperformed the control, respectively (OR 0.80, 95% CI 0.75‐0.86; OR=0.66, 95% CI 0.61‐0.71). Refer to Table 3 for more details on the logistic regression model of the activation rate.

Given these results, we can reject the null hypothesis for each of hypotheses 1a, 1b, 2a and 2b. We fail to reject the null hypothesis for all of hypotheses 3a, 3b, 4a, and 4b. Note that the pseudo *R^2^* for each of the click-through rate and activation rate models was less than .01, indicating most of the variance in the dependent variable for the respective model is unexplained by the independent variables.

**Table 3.** Odds ratio (OR), coefficient, standard error, and *P* value from logistic regression of activation rate.

	OR (95% CI)	Coefficient	SE	*P *value
Constant	0.01 (0.005-0.006)	–5.193	0.03	<.001
Experimental condition				
Control	*—^a^*	*—*	*—*	*—*
Condition 2	1.55 (1.45-1.65)	0.437	0.03	<.001
Condition 3	1.38 (1.29-1.47)	0.321	0.03	<.001
Condition 4	0.80 (0.75-0.86)	–0.217	0.04	<.001
Condition 5	0.66 (0.61-0.71)	–0.422	0.04	<.001
Age group (years)				
18‐24	—	—	—	—
25‐34	1.21 (1.15-1.28)	0.193	0.03	<.001
35‐44	1.56 (1.47-1.65)	0.444	0.03	<.001
45‐54	1.80 (1.69-1.93)	0.590	0.03	<.001
55+	2.01 (1.86-2.16)	0.697	0.04	<.001
Gender identification				
Female	0.97 (0.93-1.01)	–0.028	0.02	<.001
Male	—	—	—	—
Other	1.08 (0.94-1.24)	0.074	0.07	<.001

a Not applicable.

### Content Retention Post Hoc Analysis

As a post hoc exploration, we explored the differences in the 7-day retention rate with the meditation content between conditions. 7-day retention rate is defined as the percentage of people in each cell who listened to the content 7 days or more after the initial message was delivered. We would not expect a single message to necessarily drive long-term retention with the content, and therefore chose not to include it as a primary objective for the larger experiment. The *χ*^2^ test for the 7-day retention rate across all conditions yielded *χ*^2^_4_=33.46 (*P*<.001), indicating significant differences in retention across conditions.

Like the click-through and activation rates, to assess the effect size differences across conditions, we conducted a logistic regression using the 7 day retention rate as the dependent variable. Each of the experimental conditions, as well as the age bins and gender variables used in the previous models, served as the independent variables in this model. In doing so, Condition 2 no longer had an effect that was statistically different from the control (OR 1.21, 95% CI 0.95‐1.55). Condition 3 was higher than the control, but with a confidence interval lower bound close to one (OR 1.29, 95% CI 1.01‐1.64). Retention in Condition 4 remained lower than the control (OR 0.69, 95% CI 0.53‐0.91), while Condition 5 was not (OR 0.88, 95% CI 0.68‐1.14). See Table 4 for more details on the logistic regression model of the 7-day retention rate. Like the other models, the pseudo *R^2^* for the retention rate model was less than .01, indicating most of the variance in retention is unexplained by the independent variables.

**Table 4.** Odds ratio (OR), coefficient, SE, and *P* value from logistic regression of post-7 day retention rate.

	OR (95% CI)	Coefficient	SE	*P* value
Constant	0.00 (0.00-0.00)	–7.913	0.13	<.001
Experimental condition				
Control	*—^a^*	*—*	*—*	*—*
Condition 2	1.21 (0.95-1.55)	0.194	0.12	.11
Condition 3	1.29 (1.01-1.64)	0.253	0.12	.04
Condition 4	0.69 (0.53-0.91)	–0.368	0.14	.01
Condition 5	0.88 (0.68-1.14)	–0.127	0.13	.34
Age group, (years)				
18‐24	—	—	—	—
25‐34	1.37 (1.12-1.68)	0.317	0.10	<.001
35‐44	1.57 (1.25-1.97)	0.451	0.12	<.001
45‐54	2.01 (1.55-2.61)	0.700	0.13	<.001
55+	2.33 (1.76-3.09)	0.847	0.14	<.001
Gender identification				
Female	1.01 (0.87-1.18)	0.013	0.08	.86
Male	—	—	—	—
Other	0.91 (0.51-1.62)	–0.096	0.29	.74

a Not applicable.

## Discussion

### Principal Results

This research aimed to determine whether the application of theory-based messages designed to break down specific barriers to meditation could serve as an effective entry point that encouraged people to explore meditation content. While the outcomes were mixed, in that some messages outperformed the control while others underperformed, the results offer useful insights into the complexities of health communication within the context of meditation. The gaps in performance across messages, specifically that the best theory-based message performed substantively better than the worst theory-based message, underscore the critical role of message framing in health communication strategies for meditation.

We believe that the field experiment methodology, coupled with very large sample sizes, is a strength of the study. While the study has a great deal of statistical power, the differences in effect size between conditions are meaningful and clinically useful, particularly in the field of health behavior change. For example, in a meta-analysis of the Theory of Planned Behavior, McEachan et al [22] found that the theory only predicted about 12.1% of the variance in behavior change for physical activity using objective measures in controlled, research environments. With that little variance explained in controlled research environments, we believe that increases of 1.55 in the OR of people engaging with content based on differences in communication are meaningful and of clinical importance. Furthermore, our findings are in line with recent public health research that found social media health advertising changed around 1% of viewer attitudes, providing meaningful and cost-effective tools for positive changes in public health [23].

Condition 2, which performed best, directly challenged the barrier outlined by Hunt et al [16] and Williams et al [15] about the need for quiet and solitude in meditation. The message highlighted that to meditate, one does not necessarily require silence (such as meditating in public, during walking meditation, and other similar practices). Breaking down this barrier may have boosted self-efficacy [17] by aligning the practice with more accessible, everyday settings that fit into people’s busy lifestyles, thus enhancing their belief in their ability to find time for meditation. This finding offers compelling evidence for future researchers who study meditation engagement that it is worthwhile to explore ways to enable meditators to integrate the practice into their everyday lives.

Conversely, Conditions 4 and 5, which addressed the social stigma (such as people thinking one is strange for engaging in meditation) and lack of knowledge about meditation (such as not knowing whether they are doing it correctly), did not perform as well as even the generic control message. They may have failed to resonate because they did not sufficiently assure people of their capability to meditate successfully amidst the perceived obstacles, which is a crucial aspect of self-efficacy [17]. It is also possible that these particular barriers are less salient to the target sample or that the language used in the messages was unappealing or interpreted negatively.

In addition, the post hoc analysis revealed that while there were differences between messages based on the click-through rate and the activation rate, the differences significantly diminished when we assessed retention with the content past day 7 after the message was viewed. This indicates that a single message, even when designed to explicitly break down a substantive barrier to meditation, may not necessarily be effective in driving long-term behavior change with meditation. However, the meditation content itself may not have effectively engaged people who activated, and subsequently, it was not an issue with the messaging, but the content.

From a practical perspective, this research provides robust, empirically validated insights that meditation program managers and content providers can use in their own communication strategies. Those messages that proved more effective than the control can either be repurposed directly or revised appropriately as a starting point for meditation messaging strategy.

### Comparison With Prior Work

We grounded this study in SCT, particularly focusing on self-efficacy and perceived barriers to meditation [17]. This aligns with many health communication interventions that use established psychological models to structure message design. Fishbein and Cappella [24] emphasized the importance of grounding persuasive health messages in behavioral theory, including SCT. Similarly, Arguel et al [25] found that most digital health interventions rely on self-efficacy, motivation, and behavioral control models to drive engagement. Moreover, many digital health interventions emphasize barrier reduction and efficacy enhancement [26]. Prior studies have also demonstrated that theory-based messages generally improve engagement compared to generic messages, a trend consistently observed in health communication research [24]. However, our study revealed that not all theoretically driven messages worked as expected. Specifically, the knowledge-focused message performed worse than the control, challenging the assumption that providing information alone improves behavior adoption. Similarly, a message addressing a sociocultural barrier, an environmental factor in SCT [27], did not outperform the generic message, suggesting that social influence may not be a key determinant of meditation behavior change.

We conjecture that knowledge-based (Condition 5) and social influence-based interventions (Condition 4) were less effective than generic messages because people may hesitate to practice meditation for reasons unrelated to knowledge gaps or social influence. In other words, people might not perceive meditation as inherently difficult, but rather as constrained by practical barriers (Condition 2) or a lack of perceived benefits (Condition 3). Similarly, it is plausible that people view meditation as an individual, private practice, meaning that social influence (eg, how others perceive their meditation practice) is not a strong motivating factor. These are plausible explanations, not confirmed causal mechanisms, and require further exploration to be substantiated.

A key implication of our findings is that different barriers influence self-efficacy in distinct ways. While some barriers, such as practical constraints and perceived benefits, may directly impact confidence in one’s ability to engage in meditation, others, like social influence, may have weaker or more indirect effects. This suggests that interventions aiming to enhance self-efficacy should carefully consider which barriers they target and how they tailor messages to address them. Future work should examine how different types of barriers interact with self-efficacy and whether tailoring messages to individuals’ perceived barriers leads to more effective behavior change.

### Limitations

There are also important limitations to this research to consider. First, despite the messages that were included in this research being based on specific barriers to meditation, the messages could have been articulated in many different ways. The study design could have included more messages to control for these differences, such as different articulations of the same barrier or combinations of different barriers. While the results here showed clear differences in performance across messages, other approaches to crafting the messages may have performed differently. In addition, the word count and Flesch Reading Ease Score for the messages vary and could ostensibly explain some of the differences in the performance of the messages. However, because these measures of message complexity are perfectly collinear with the message conditions themselves, we could not control for these differences across messages in the model directly.

In addition, the initial sample for this research was larger than the 1.33 million people who were exposed to one of the experimental conditions. The reason some people in the target sample were not exposed to the message is because they may not have used the app or visited the Spotify Home page in the app during the fieldwork time period, and therefore were not included in the study. The messaging system used for this study also delivered somewhat fewer messages to users in the control condition, compared to the test conditions. While the control condition had fewer total people than the test conditions (albeit still well over one hundred thousand), there is no evidence of a systematic bias that would alter the outcomes, and comparisons between the individual test conditions demonstrate substantive differences between messages.

Finally, this study aimed to break down the barriers to meditation and subsequently increase self-efficacy to engage in the practice, but we did not test self-efficacy in the model directly, nor did we pretest the messages to assess whether they did indeed increase self-efficacy. While breaking down barriers is generally understood to increase self-efficacy, the messages may have failed to effectively do so.

### Future Directions

Hunt et al [16] found that pragmatic challenges to meditating, such as finding time or a quiet place, are a substantive barrier to the practice. This research uncovered that addressing this barrier through messaging was a more effective strategy to encourage people to explore and experiment with meditation content than addressing other barriers. Considering this, future researchers who explore meditation habit formation may want to consider focusing their efforts on how to educate people about engaging in meditation during their daily activities to more effectively fit the practice into their busy lifestyles.

Future research might also more deeply explore the novelty effect of health communication messages. In the current study, the most effective condition may have provided participants with new, novel information compared to other conditions, but this dimension was not directly controlled for in the experimental design.

This research also explored whether breaking down barriers to meditation compels people to explore and experiment with meditation content on a digital platform, but the role of people exploring new meditation content and experimenting with it, particularly in the larger context of developing a meditation habit, is unclear. Future research might assess the role of meditation content exploration and experimentation with meditation generally to better understand how that fits into the broader context of developing or sustaining a meditation practice.

Finally, this research only included a single platform, Spotify, so there is a need to further validate the conclusions by testing on other platforms and other environments.

### Conclusion

Meditation has potential health benefits for many people, and while people are generally aware of what it is, few people have an established meditation practice. The theoretical barriers to meditation are generally understood, but to the authors’ knowledge, there has been no real-world application of this knowledge to explore whether these barriers can be effectively broken down. This research provides compelling evidence that breaking down the barriers to meditation can be effective in encouraging people to explore meditation content, but not universally so. In addition, this type of messaging may result in short-term engagement, but may not lead to longer-term behavior change. Finally, this research suggests that educating and encouraging people to engage in meditation during their daily activities may be an effective means to encourage meditation.

## Abbreviations

IRB: institutional review board
OR: odds ratio
SCT: social cognitive theory

## References

[1] Lam SU, Riordan KM, Simonsson O, Davidson RJ, Goldberg SB (2023). Who sticks with meditation? Rates and predictors of persistence in a population-based sample in the usa. Mindfulness (N Y).

[2] Pepping CA, Walters B, Davis PJ, O’Donovan A (2016). Why do people practice mindfulness? An investigation into reasons for practicing mindfulness meditation. Mindfulness (N Y).

[3] Sedlmeier P, Theumer J (2020). Why do people begin to meditate and why do they continue?. Mindfulness (N Y).

[4] Huberty J, Vranceanu AM, Carney C, Breus M, Gordon M, Puzia ME (2019). Characteristics and usage patterns among 12,151 paid subscribers of the calm meditation app: Cross-sectional survey. JMIR Mhealth Uhealth.

[5] Macinko J, Upchurch DM (2019). Factors associated with the use of meditation, U.S. adults 2017. J Altern Complement Med.

[6] Cramer H, Hall H, Leach M (2016). Prevalence, patterns, and predictors of meditation use among US adults: a nationally representative survey. Sci Rep.

[7] Anderson T, Suresh M, Farb NA (2019). Meditation benefits and drawbacks: empirical codebook and implications for teaching. J Cogn Enhanc.

[8] Davis KM, Wojcik CM, Baillie AJ (2024). Mechanisms of mindfulness: a longitudinal study of a mindfulness-based stress reduction program. Mindfulness (N Y).

[9] Fincham GW, Mavor K, Dritschel B (2023). Effects of mindfulness meditation duration and type on well-being: an online dose-ranging randomized controlled trial. Mindfulness (N Y).

[10] Galante J, Grabovac A, Wright M (2023). A framework for the empirical investigation of mindfulness meditative development. Mindfulness (N Y).

[11] Goldberg SB, Tucker RP, Greene PA (2018). Mindfulness-based interventions for psychiatric disorders: a systematic review and meta-analysis. Clin Psychol Rev.

[12] Bowen M, Beam M Mapping mindfulness: assessing the stages of meditation habit formation in the USA using the Sussex Mindfulness Meditation (SuMMed) model. Mindfulness (N Y).

[13] Lam SU, Xie Q, Goldberg SB (2023). Situating meditation apps within the ecosystem of meditation practice: population-based survey study. JMIR Ment Health.

[14] Baumel A, Muench F, Edan S, Kane JM (2019). Objective user engagement with mental health apps: systematic search and panel-based usage analysis. J Med Internet Res.

[15] Williams AL, Dixon J, McCorkle R, Van Ness PH (2011). Determinants of meditation practice inventory: development, content validation, and initial psychometric testing. Altern Ther Health Med.

[16] Hunt CA, Hoffman MA, Mohr JJ, Williams AL (2020). Assessing perceived barriers to meditation: the Determinants of Meditation Practice Inventory-Revised (DMPI-R). Mindfulness (N Y).

[17] Bandura A (1977). Self-efficacy: toward a unifying theory of behavioral change. Psychol Rev.

[18] Abraham C, Michie S (2008). A taxonomy of behavior change techniques used in interventions. Health Psychol.

[19] Worrall AP, Connolly MJ, O’Neill A (2020). Readability of online COVID-19 health information: a comparison between four English speaking countries. BMC Public Health.

[20] (2025). Textstat. Python Package Index.

[21] (2025). Statsmodels.

[22] McEachan RRC, Conner M, Taylor NJ, Lawton RJ (2011). Prospective prediction of health-related behaviours with the Theory of Planned Behaviour: a meta-analysis. Health Psychol Rev.

[23] Athey S, Grabarz K, Luca M, Wernerfelt N (2023). Digital public health interventions at scale: the impact of social media advertising on beliefs and outcomes related to COVID vaccines. Proc Natl Acad Sci U S A.

[24] Fishbein M, Cappella JN (2006). The role of theory in developing effective health communications. J Commun.

[25] Arguel A, Perez‐Concha O, Li SYW, Lau AYS (2018). Theoretical approaches of online social network interventions and implications for behavioral change: a systematic review. J Eval Clin Pract.

[26] Webb TL, Joseph J, Yardley L, Michie S (2010). Using the internet to promote health behavior change: a systematic review and meta-analysis of the impact of theoretical basis, use of behavior change techniques, and mode of delivery on efficacy. J Med Internet Res.

[27] Bandura A (1998). Health promotion from the perspective of social cognitive theory. Psychology & Health.

